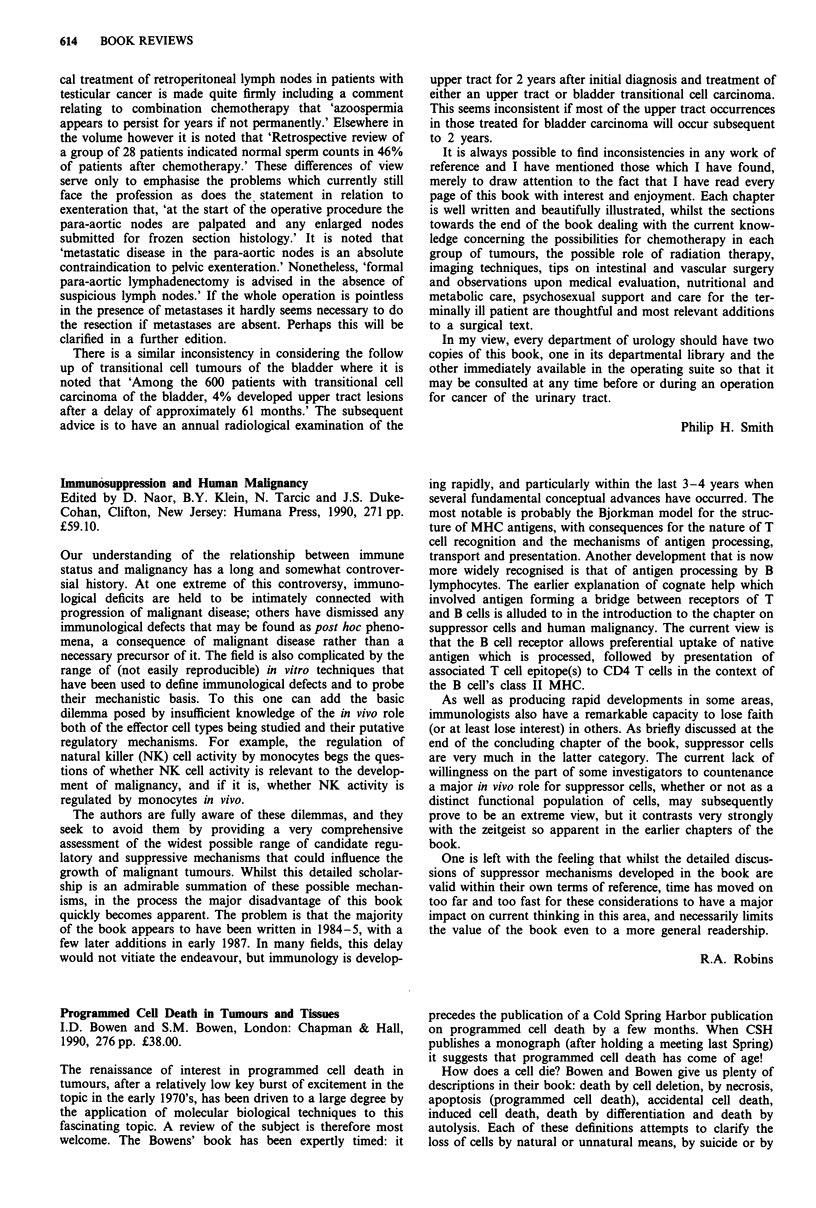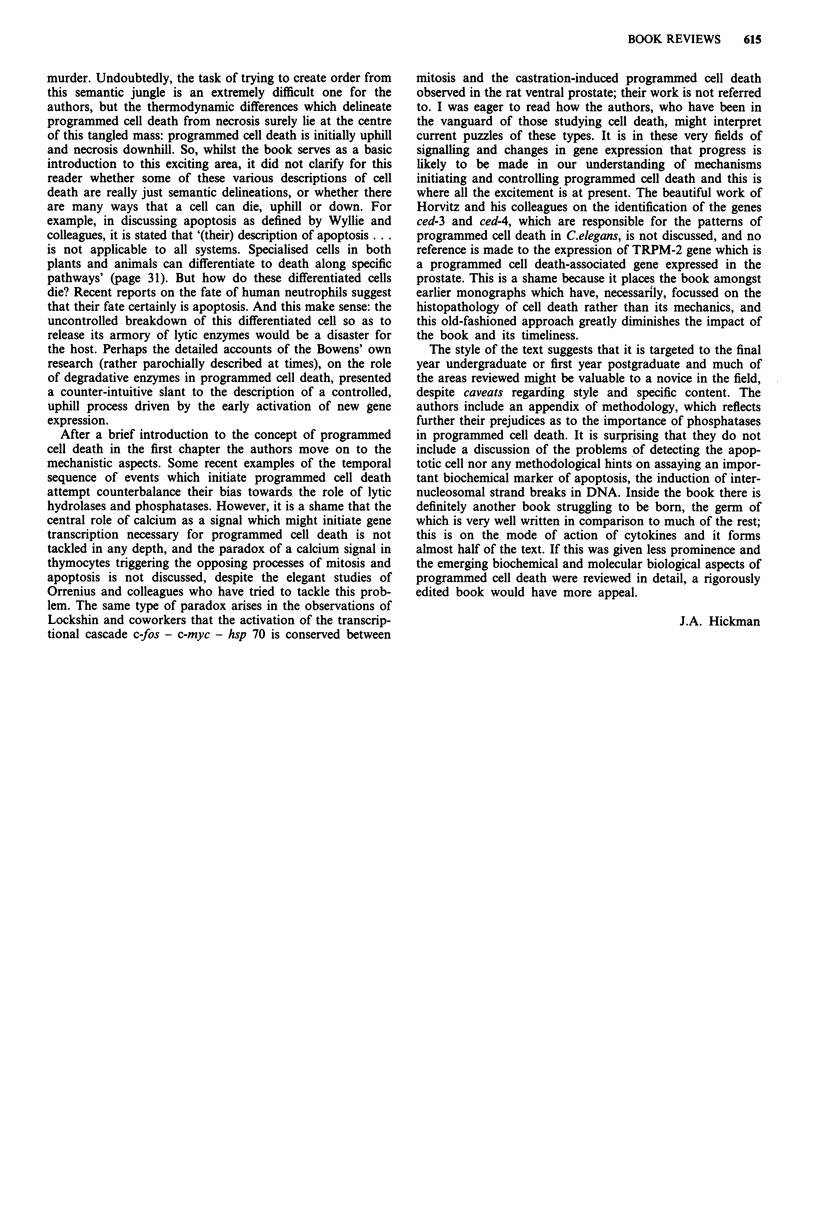# Programmed Cell Death in Tumours and Tissues

**Published:** 1991-09

**Authors:** J.A. Hickman


**Programmed Cell Death in Tumours and Tissues**
I.D. Bowen and S.M. Bowen, London: Chapman & Hall, 1990, 276 pp. £38.00.

The renaissance of interest in programmed cell death in tumours, after a relatively low key burst of excitement in the topic in the early 1970's, has been driven to a large degree by the application of molecular biological techniques to this fascinating topic. A review of the subject is therefore most welcome. The Bowens' book has been expertly timed: it precedes the publication of a Cold Spring Harbor publication on programmed cell death by a few months. When CSH publishes a monograph (after holding a meeting last Spring) it suggests that programmed cell death has come of age!

How does a cell die? Bowen and Bowen give us plenty of descriptions in their book: death by cell deletion, by necrosis, apoptosis (programmed cell death), accidental cell death, induced cell death, death by differentiation and death by autolysis. Each of these definitions attempts to clarify the loss of cells by natural or unnatural means, by suicide or by murder. Undoubtedly, the task of trying to create order from this semantic jungle is an extremely difficult one for the authors, but the thermodynamic differences which delineate programmed cell death from necrosis surely lie at the centre of this tangled mass: programmed cell death is initially uphill and necrosis downhill. So, whilst the book serves as a basic introduction to this exciting area, it did not clarify for this reader whether some of these various descriptions of cell death are really just semantic delineations, or whether there are many ways that a cell can die, uphill or down. For example, in discussing apoptosis as defined by Wyllie and colleagues, it is stated that '(their) description of apoptosis . . . is not applicable to all systems. Specialised cells in both plants and animals can differentiate to death along specific pathways' (page 31). But how do these differentiated cells die? Recent reports on the fate of human neutrophils suggest that their fate certainly is apoptosis. And this make sense: the uncontrolled breakdown of this differentiated cell so as to release its armory of lytic enzymes would be a disaster for the host. Perhaps the detailed accounts of the Bowens' own research (rather parochially described at times), on the role of degradative enzymes in programmed cell death, presented a counter-intuitive slant to the description of a controlled, uphill process driven by the early activation of new gene expression.

After a brief introduction to the concept of programmed cell death in the first chapter the authors move on to the mechanistic aspects. Some recent examples of the temporal sequence of events which initiate programmed cell death attempt counterbalance their bias towards the role of lytic hydrolases and phosphatases. However, it is a shame that the central role of calcium as a signal which might initiate gene transcription necessary for programmed cell death is not tackled in any depth, and the paradox of a calcium signal in thymocytes triggering the opposing processes of mitosis and apoptosis is not discussed, despite the elegant studies of Orrenius and colleagues who have tried to tackle this problem. The same type of paradox arises in the observations of Lockshin and coworkers that the activation of the transcriptional cascade c-*fos* – c-*myc* – *hsp* 70 is conserved between mitosis and the castration-induced programmed cell death observed in the rat ventral prostate; their work is not referred to. I was eager to read how the authors, who have been in the vanguard of those studying cell death, might interpret current puzzles of these types. It is in these very fields of signalling and changes in gene expression that progress is likely to be made in our understanding of mechanisms initiating and controlling programmed cell death and this is where all the excitement is at present. The beautiful work of Horvitz and his colleagues on the identification of the genes *ced*-3 and *ced*-4, which are responsible for the patterns of programmed cell death in *C.elegans*, is not discussed, and no reference is made to the expression of TRPM-2 gene which is a programmed cell death-associated gene expressed in the prostate. This is a shame because it places the book amongst earlier monographs which have, necessarily, focussed on the histopathology of cell death rather than its mechanics, and this old-fashioned approach greatly diminishes the impact of the book and its timeliness.

The style of the text suggests that it is targeted to the final year undergraduate or first year postgraduate and much of the areas reviewed might be valuable to a novice in the field, despite *caveats* regarding style and specific content. The authors include an appendix of methodology, which reflects further their prejudices as to the importance of phosphatases in programmed cell death. It is surprising that they do not include a discussion of the problems of detecting the apoptotic cell nor any methodological hints on assaying an important biochemical marker of apoptosis, the induction of internucleosomal strand breaks in DNA. Inside the book there is definitely another book struggling to be born, the germ of which is very well written in comparison to much of the rest; this is on the mode of action of cytokines and it forms almost half of the text. If this was given less prominence and the emerging biochemical and molecular biological aspects of programmed cell death were reviewed in detail, a rigorously edited book would have more appeal.

J.A. Hickman